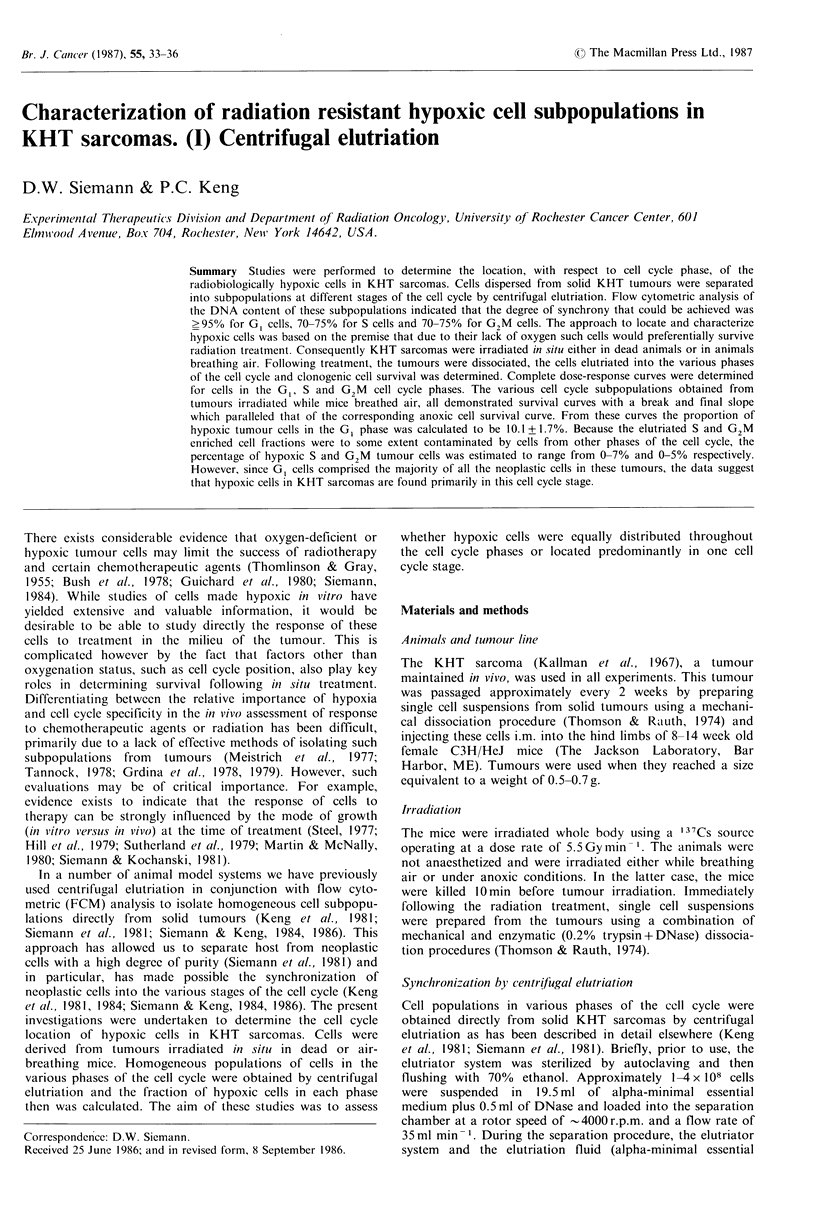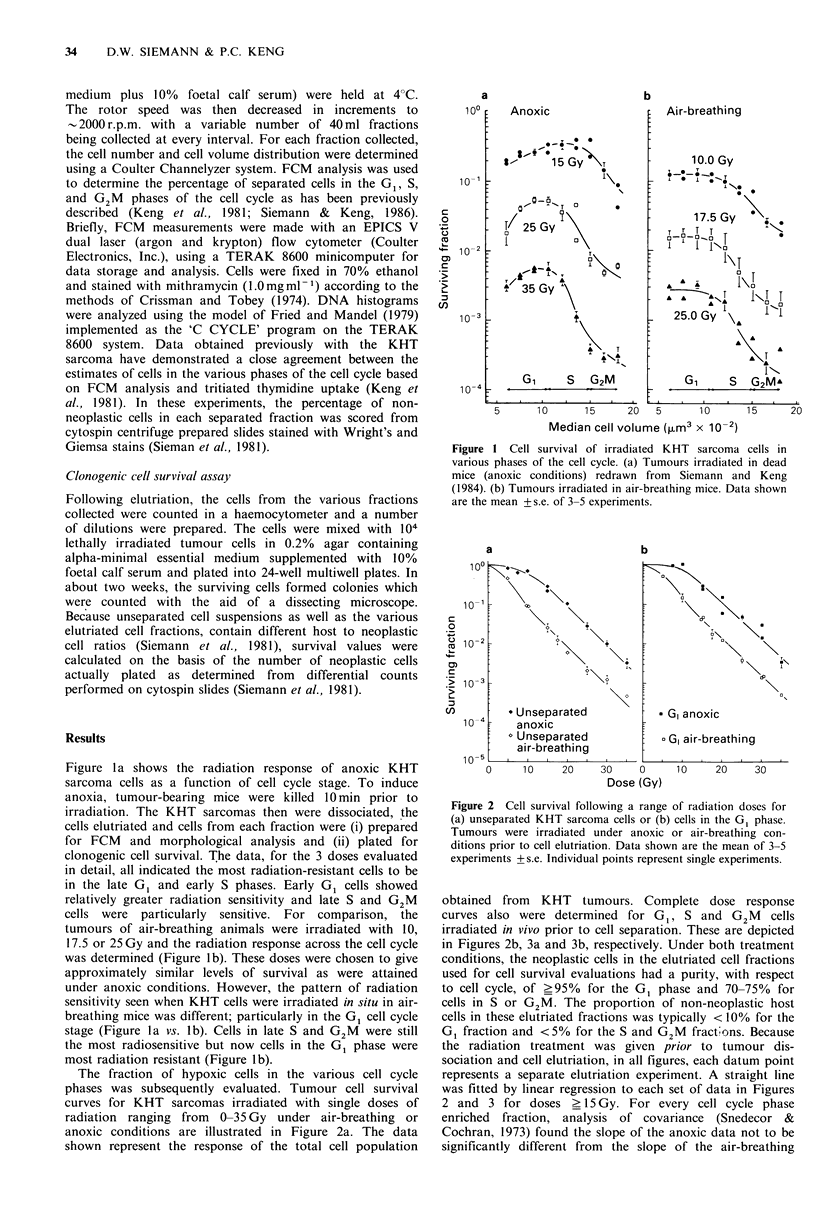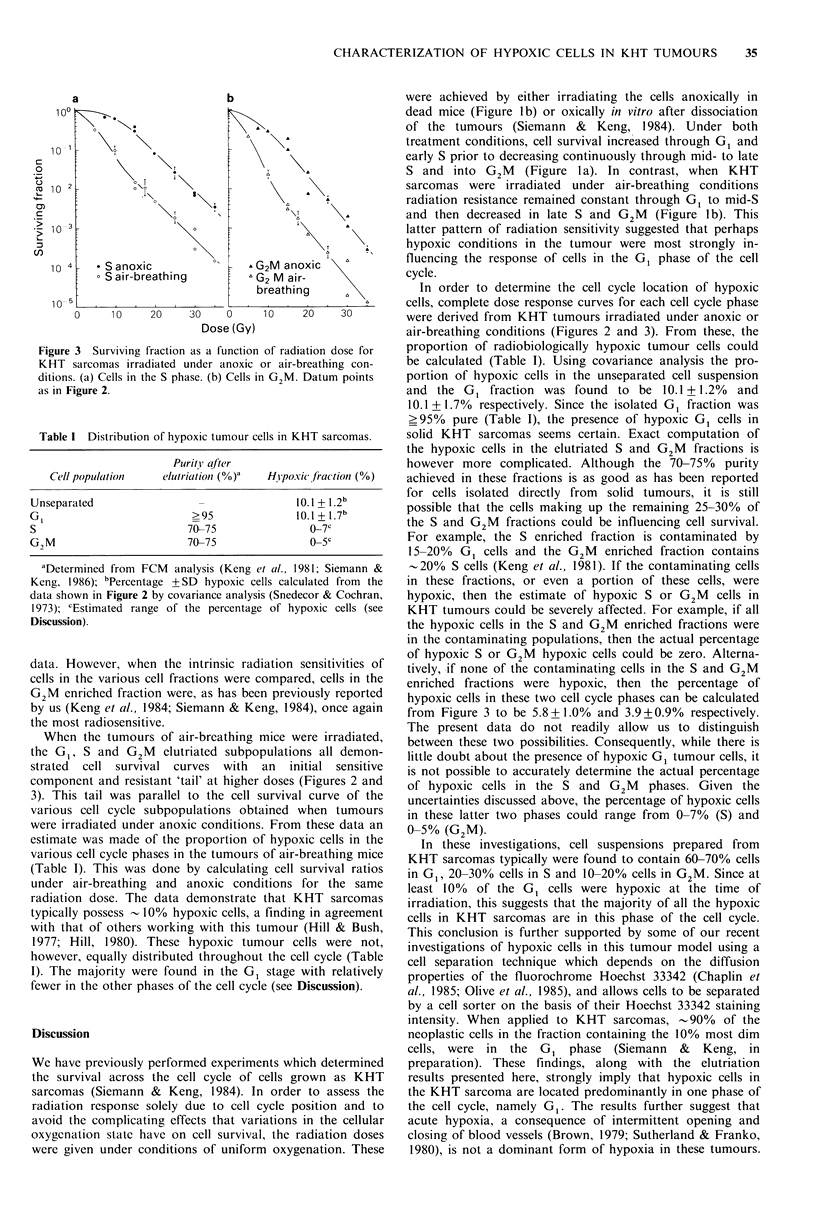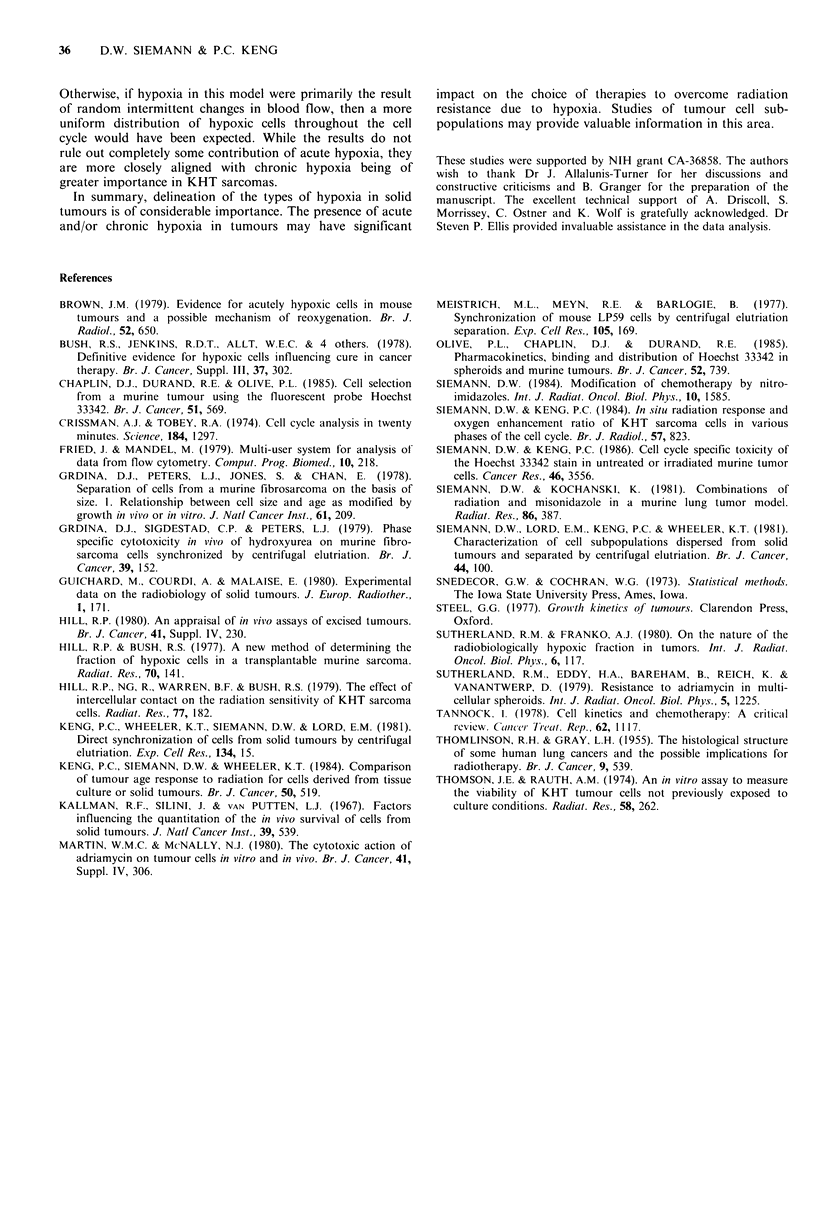# Characterization of radiation resistant hypoxic cell subpopulations in KHT sarcomas. (I). Centrifugal elutriation.

**DOI:** 10.1038/bjc.1987.7

**Published:** 1987-01

**Authors:** D. W. Siemann, P. C. Keng

## Abstract

Studies were performed to determine the location, with respect to cell cycle phase, of the radiobiologically hypoxic cells in KHT sarcomas. Cells dispersed from solid KHT tumours were separated into subpopulations at different stages of the cell cycle by centrifugal elutriation. Flow cytometric analysis of the DNA content of these subpopulations indicated that the degree of synchrony that could be achieved was greater than or equal to 95% for G1 cells, 70-75% for S cells and 70-75% for G2M cells. The approach to locate and characterize hypoxic cells was based on the premise that due to their lack of oxygen such cells would preferentially survive radiation treatment. Consequently KHT sarcomas were irradiated in situ either in dead animals or in animals breathing air. Following treatment, the tumours were dissociated, the cells elutriated into the various phases of the cell cycle and clonogenic cell survival was determined. Complete dose-response curves were determined for cells in the G1, S and G2M cell cycle phases. The various cell cycle subpopulations obtained from tumours irradiated while mice breathed air, all demonstrated survival curves with a break and final slope which paralleled that of the corresponding anoxic cell survival curve. From these curves the proportion of hypoxic tumour cells in the G1 phase was calculated to be 10.1 +/- 1.7%. Because the elutriated S and G2M enriched cell fractions were to some extent contaminated by cells from other phases of the cell cycle, the percentage of hypoxic S and G2M tumour cells was estimated to range from 0-7% and 0-5% respectively. However, since G1 cells comprised the majority of all the neoplastic cells in these tumours, the data suggest that hypoxic cells in KHT sarcomas are found primarily in this cell cycle stage.


Br. J. Cancer (1987), 55, 33–36 © The Macmillan Press Ltd., 1987

### Characterization of radiation resistant hypoxic cell subpopulations in KHT sarcomas. (I) Centrifugal elutriation

D.W. Siemann & P.C. Keng

*Experimental Therapeutics Division and Department of Radiation Oncology, University of Rochester Cancer Center, 601 Elmwood Avenue, Box 704, Rochester, New York 14642, USA.*

**Summary** Studies were performed to determine the location, with respect to cell cycle phase, of the radiobiologically hypoxic cells in KHT sarcomas. Cells dispersed from solid KHT tumours were separated into subpopulations at different stages of the cell cycle by centrifugal elutriation. Flow cytometric analysis of the DNA content of these subpopulations indicated that the degree of synchrony that could be achieved was ≥95% for $G_1$ cells, 70–75% for S cells and 70–75% for $G_2M$ cells. The approach to locate and characterize hypoxic cells was based on the premise that due to their lack of oxygen such cells would preferentially survive radiation treatment. Consequently KHT sarcomas were irradiated *in situ* either in dead animals or in animals breathing air. Following treatment, the tumours were dissociated, the cells elutriated into the various phases of the cell cycle and clonogenic cell survival was determined. Complete dose-response curves were determined for cells in the $G_1$, S and $G_2M$ cell cycle phases. The various cell cycle subpopulations obtained from tumours irradiated while mice breathed air, all demonstrated survival curves with a break and final slope which paralleled that of the corresponding anoxic cell survival curve. From these curves the proportion of hypoxic tumour cells in the $G_1$ phase was calculated to be $10.1 \pm 1.7\%$. Because the elutriated S and $G_2M$ enriched cell fractions were to some extent contaminated by cells from other phases of the cell cycle, the percentage of hypoxic S and $G_2M$ tumour cells was estimated to range from 0–7% and 0–5% respectively. However, since $G_1$ cells comprised the majority of all the neoplastic cells in these tumours, the data suggest that hypoxic cells in KHT sarcomas are found primarily in this cell cycle stage.

There exists considerable evidence that oxygen-deficient or hypoxic tumour cells may limit the success of radiotherapy and certain chemotherapeutic agents (Thomlinson & Gray, 1955; Bush *et al.*, 1978; Guichard *et al.*, 1980; Siemann, 1984). While studies of cells made hypoxic *in vitro* have yielded extensive and valuable information, it would be desirable to be able to study directly the response of these cells to treatment in the milieu of the tumour. This is complicated however by the fact that factors other than oxygenation status, such as cell cycle position, also play key roles in determining survival following *in situ* treatment. Differentiating between the relative importance of hypoxia and cell cycle specificity in the *in vivo* assessment of response to chemotherapeutic agents or radiation has been difficult, primarily due to a lack of effective methods of isolating such subpopulations from tumours (Meistrich *et al.*, 1977; Tannock, 1978; Grdina *et al.*, 1978, 1979). However, such evaluations may be of critical importance. For example, evidence exists to indicate that the response of cells to therapy can be strongly influenced by the mode of growth (*in vitro versus in vivo*) at the time of treatment (Steel, 1977; Hill *et al.*, 1979; Sutherland *et al.*, 1979; Martin & McNally, 1980; Siemann & Kochanski, 1981).

In a number of animal model systems we have previously used centrifugal elutriation in conjunction with flow cytometric (FCM) analysis to isolate homogeneous cell subpopulations directly from solid tumours (Keng *et al.*, 1981; Siemann *et al.*, 1981; Siemann & Keng, 1984, 1986). This approach has allowed us to separate host from neoplastic cells with a high degree of purity (Siemann *et al.*, 1981) and in particular, has made possible the synchronization of neoplastic cells into the various stages of the cell cycle (Keng *et al.*, 1981, 1984; Siemann & Keng, 1984, 1986). The present investigations were undertaken to determine the cell cycle location of hypoxic cells in KHT sarcomas. Cells were derived from tumours irradiated *in situ* in dead or air-breathing mice. Homogeneous populations of cells in the various phases of the cell cycle were obtained by centrifugal elutriation and the fraction of hypoxic cells in each phase then was calculated. The aim of these studies was to assess whether hypoxic cells were equally distributed throughout the cell cycle phases or located predominantly in one cell cycle stage.

Correspondence: D.W. Siemann.
Received 25 June 1986; and in revised form, 8 September 1986.

### Materials and methods

### Animals and tumour line

The KHT sarcoma (Kallman *et al.*, 1967), a tumour maintained *in vivo*, was used in all experiments. This tumour was passaged approximately every 2 weeks by preparing single cell suspensions from solid tumours using a mechanical dissociation procedure (Thomson & Rauth, 1974) and injecting these cells i.m. into the hind limbs of 8–14 week old female C3H/HeJ mice (The Jackson Laboratory, Bar Harbor, ME). Tumours were used when they reached a size equivalent to a weight of 0.5–0.7 g.

### Irradiation

The mice were irradiated whole body using a $^{137}Cs$ source operating at a dose rate of $5.5\,Gy\,min^{-1}$. The animals were not anaesthetized and were irradiated either while breathing air or under anoxic conditions. In the latter case, the mice were killed 10 min before tumour irradiation. Immediately following the radiation treatment, single cell suspensions were prepared from the tumours using a combination of mechanical and enzymatic (0.2% trypsin + DNase) dissociation procedures (Thomson & Rauth, 1974).

### Synchronization by centrifugal elutriation

Cell populations in various phases of the cell cycle were obtained directly from solid KHT sarcomas by centrifugal elutriation as has been described in detail elsewhere (Keng *et al.*, 1981; Siemann *et al.*, 1981). Briefly, prior to use, the elutriator system was sterilized by autoclaving and then flushing with 70% ethanol. Approximately $1\text{–}4 \times 10^8$ cells were suspended in 19.5 ml of alpha-minimal essential medium plus 0.5 ml of DNase and loaded into the separation chamber at a rotor speed of ~4000 r.p.m. and a flow rate of $35\,ml\,min^{-1}$. During the separation procedure, the elutriator system and the elutriation fluid (alpha-minimal essential medium plus 10% foetal calf serum) were held at 4°C. The rotor speed was then decreased in increments to ~2000 r.p.m. with a variable number of 40 ml fractions being collected at every interval. For each fraction collected, the cell number and cell volume distribution were determined using a Coulter Channelyzer system. FCM analysis was used to determine the percentage of separated cells in the $G_1$, S, and $G_2M$ phases of the cell cycle as has been previously described (Keng *et al.*, 1981; Siemann & Keng, 1986). Briefly, FCM measurements were made with an EPICS V dual laser (argon and krypton) flow cytometer (Coulter Electronics, Inc.), using a TERAK 8600 minicomputer for data storage and analysis. Cells were fixed in 70% ethanol and stained with mithramycin ($1.0\,mg\,ml^{-1}$) according to the methods of Crissman and Tobey (1974). DNA histograms were analyzed using the model of Fried and Mandel (1979) implemented as the 'C CYCLE' program on the TERAK 8600 system. Data obtained previously with the KHT sarcoma have demonstrated a close agreement between the estimates of cells in the various phases of the cell cycle based on FCM analysis and tritiated thymidine uptake (Keng *et al.*, 1981). In these experiments, the percentage of non-neoplastic cells in each separated fraction was scored from cytospin centrifuge prepared slides stained with Wright's and Giemsa stains (Sieman *et al.*, 1981).

*Clonogenic cell survival assay*

Following elutriation, the cells from the various fractions collected were counted in a haemocytometer and a number of dilutions were prepared. The cells were mixed with $10^4$ lethally irradiated tumour cells in 0.2% agar containing alpha-minimal essential medium supplemented with 10% foetal calf serum and plated into 24-well multiwell plates. In about two weeks, the surviving cells formed colonies which were counted with the aid of a dissecting microscope. Because unseparated cell suspensions as well as the various elutriated cell fractions, contain different host to neoplastic cell ratios (Siemann *et al.*, 1981), survival values were calculated on the basis of the number of neoplastic cells actually plated as determined from differential counts performed on cytospin slides (Siemann *et al.*, 1981).

### Results

Figure 1a shows the radiation response of anoxic KHT sarcoma cells as a function of cell cycle stage. To induce anoxia, tumour-bearing mice were killed 10 min prior to irradiation. The KHT sarcomas then were dissociated, the cells elutriated and cells from each fraction were (i) prepared for FCM and morphological analysis and (ii) plated for clonogenic cell survival. The data, for the 3 doses evaluated in detail, all indicated the most radiation-resistant cells to be in the late $G_1$ and early S phases. Early $G_1$ cells showed relatively greater radiation sensitivity and late S and $G_2M$ cells were particularly sensitive. For comparison, the tumours of air-breathing animals were irradiated with 10, 17.5 or 25 Gy and the radiation response across the cell cycle was determined (Figure 1b). These doses were chosen to give approximately similar levels of survival as were attained under anoxic conditions. However, the pattern of radiation sensitivity seen when KHT cells were irradiated *in situ* in air-breathing mice was different; particularly in the $G_1$ cell cycle stage (Figure 1a *vs.* 1b). Cells in late S and $G_2M$ were still the most radiosensitive but now cells in the $G_1$ phase were most radiation resistant (Figure 1b).

The fraction of hypoxic cells in the various cell cycle phases was subsequently evaluated. Tumour cell survival curves for KHT sarcomas irradiated with single doses of radiation ranging from 0–35 Gy under air-breathing or anoxic conditions are illustrated in Figure 2a. The data shown represent the response of the total cell population

**Figure 1** Cell survival of irradiated KHT sarcoma cells in various phases of the cell cycle. (a) Tumours irradiated in dead mice (anoxic conditions) redrawn from Siemann and Keng (1984). (b) Tumours irradiated in air-breathing mice. Data shown are the mean ±s.e. of 3–5 experiments.

**Figure 2** Cell survival following a range of radiation doses for (a) unseparated KHT sarcoma cells or (b) cells in the $G_1$ phase. Tumours were irradiated under anoxic or air-breathing conditions prior to cell elutriation. Data shown are the mean of 3–5 experiments ±s.e. Individual points represent single experiments.

obtained from KHT tumours. Complete dose response curves also were determined for $G_1$, S and $G_2M$ cells irradiated *in vivo* prior to cell separation. These are depicted in Figures 2b, 3a and 3b, respectively. Under both treatment conditions, the neoplastic cells in the elutriated cell fractions used for cell survival evaluations had a purity, with respect to cell cycle, of ≧95% for the $G_1$ phase and 70–75% for cells in S or $G_2M$. The proportion of non-neoplastic host cells in these elutriated fractions was typically <10% for the $G_1$ fraction and <5% for the S and $G_2M$ fractions. Because the radiation treatment was given *prior* to tumour dissociation and cell elutriation, in all figures, each datum point represents a separate elutriation experiment. A straight line was fitted by linear regression to each set of data in Figures 2 and 3 for doses ≧15 Gy. For every cell cycle phase enriched fraction, analysis of covariance (Snedecor & Cochran, 1973) found the slope of the anoxic data not to be significantly different from the slope of the air-breathing

**Figure 3** Surviving fraction as a function of radiation dose for KHT sarcomas irradiated under anoxic or air-breathing conditions. (a) Cells in the S phase. (b) Cells in $G_2M$. Datum points as in **Figure 2**.

**Table I** Distribution of hypoxic tumour cells in KHT sarcomas.

| *Cell population* | *Purity after elutriation* (%)[a] | *Hypoxic fraction* (%) |
|---|---|---|
| Unseparated | – | $10.1 \pm 1.2$[b] |
| $G_1$ | $\geq 95$ | $10.1 \pm 1.7$[b] |
| S | 70–75 | 0–7[c] |
| $G_2M$ | 70–75 | 0–5[c] |

[a]Determined from FCM analysis (Keng *et al.*, 1981; Siemann & Keng, 1986); [b]Percentage ±SD hypoxic cells calculated from the data shown in **Figure 2** by covariance analysis (Snedecor & Cochran, 1973); [c]Estimated range of the percentage of hypoxic cells (see **Discussion**).

data. However, when the intrinsic radiation sensitivities of cells in the various cell fractions were compared, cells in the $G_2M$ enriched fraction were, as has been previously reported by us (Keng *et al.*, 1984; Siemann & Keng, 1984), once again the most radiosensitive.

When the tumours of air-breathing mice were irradiated, the $G_1$, S and $G_2M$ elutriated subpopulations all demonstrated cell survival curves with an initial sensitive component and resistant 'tail' at higher doses (Figures 2 and 3). This tail was parallel to the cell survival curve of the various cell cycle subpopulations obtained when tumours were irradiated under anoxic conditions. From these data an estimate was made of the proportion of hypoxic cells in the various cell cycle phases in the tumours of air-breathing mice (Table I). This was done by calculating cell survival ratios under air-breathing and anoxic conditions for the same radiation dose. The data demonstrate that KHT sarcomas typically possess ~10% hypoxic cells, a finding in agreement with that of others working with this tumour (Hill & Bush, 1977; Hill, 1980). These hypoxic tumour cells were not, however, equally distributed throughout the cell cycle (Table I). The majority were found in the $G_1$ stage with relatively fewer in the other phases of the cell cycle (see **Discussion**).

### Discussion

We have previously performed experiments which determined the survival across the cell cycle of cells grown as KHT sarcomas (Siemann & Keng, 1984). In order to assess the radiation response solely due to cell cycle position and to avoid the complicating effects that variations in the cellular oxygenation state have on cell survival, the radiation doses were given under conditions of uniform oxygenation. These were achieved by either irradiating the cells anoxically in dead mice (Figure 1b) or oxically *in vitro* after dissociation of the tumours (Siemann & Keng, 1984). Under both treatment conditions, cell survival increased through $G_1$ and early S prior to decreasing continuously through mid- to late S and into $G_2M$ (Figure 1a). In contrast, when KHT sarcomas were irradiated under air-breathing conditions radiation resistance remained constant through $G_1$ to mid-S and then decreased in late S and $G_2M$ (Figure 1b). This latter pattern of radiation sensitivity suggested that perhaps hypoxic conditions in the tumour were most strongly influencing the response of cells in the $G_1$ phase of the cell cycle.

In order to determine the cell cycle location of hypoxic cells, complete dose response curves for each cell cycle phase were derived from KHT tumours irradiated under anoxic or air-breathing conditions (Figures 2 and 3). From these, the proportion of radiobiologically hypoxic tumour cells could be calculated (Table I). Using covariance analysis the proportion of hypoxic cells in the unseparated cell suspension and the $G_1$ fraction was found to be $10.1 \pm 1.2\%$ and $10.1 \pm 1.7\%$ respectively. Since the isolated $G_1$ fraction was $\geq 95\%$ pure (Table I), the presence of hypoxic $G_1$ cells in solid KHT sarcomas seems certain. Exact computation of the hypoxic cells in the elutriated S and $G_2M$ fractions is however more complicated. Although the 70–75% purity achieved in these fractions is as good as has been reported for cells isolated directly from solid tumours, it is still possible that the cells making up the remaining 25–30% of the S and $G_2M$ fractions could be influencing cell survival. For example, the S enriched fraction is contaminated by 15–20% $G_1$ cells and the $G_2M$ enriched fraction contains ~20% S cells (Keng *et al.*, 1981). If the contaminating cells in these fractions, or even a portion of these cells, were hypoxic, then the estimate of hypoxic S or $G_2M$ cells in KHT tumours could be severely affected. For example, if all the hypoxic cells in the S and $G_2M$ enriched fractions were in the contaminating populations, then the actual percentage of hypoxic S or $G_2M$ hypoxic cells could be zero. Alternatively, if none of the contaminating cells in the S and $G_2M$ enriched fractions were hypoxic, then the percentage of hypoxic cells in these two cell cycle phases can be calculated from Figure 3 to be $5.8 \pm 1.0\%$ and $3.9 \pm 0.9\%$ respectively. The present data do not readily allow us to distinguish between these two possibilities. Consequently, while there is little doubt about the presence of hypoxic $G_1$ tumour cells, it is not possible to accurately determine the actual percentage of hypoxic cells in the S and $G_2M$ phases. Given the uncertainties discussed above, the percentage of hypoxic cells in these latter two phases could range from 0–7% (S) and 0–5% ($G_2M$).

In these investigations, cell suspensions prepared from KHT sarcomas typically were found to contain 60–70% cells in $G_1$, 20–30% cells in S and 10–20% cells in $G_2M$. Since at least 10% of the $G_1$ cells were hypoxic at the time of irradiation, this suggests that the majority of all the hypoxic cells in KHT sarcomas are in this phase of the cell cycle. This conclusion is further supported by some of our recent investigations of hypoxic cells in this tumour model using a cell separation technique which depends on the diffusion properties of the fluorochrome Hoechst 33342 (Chaplin *et al.*, 1985; Olive *et al.*, 1985), and allows cells to be separated by a cell sorter on the basis of their Hoechst 33342 staining intensity. When applied to KHT sarcomas, ~90% of the neoplastic cells in the fraction containing the 10% most dim cells, were in the $G_1$ phase (Siemann & Keng, in preparation). These findings, along with the elutriation results presented here, strongly imply that hypoxic cells in the KHT sarcoma are located predominantly in one phase of the cell cycle, namely $G_1$. The results further suggest that acute hypoxia, a consequence of intermittent opening and closing of blood vessels (Brown, 1979; Sutherland & Franko, 1980), is not a dominant form of hypoxia in these tumours.

Otherwise, if hypoxia in this model were primarily the result of random intermittent changes in blood flow, then a more uniform distribution of hypoxic cells throughout the cell cycle would have been expected. While the results do not rule out completely some contribution of acute hypoxia, they are more closely aligned with chronic hypoxia being of greater importance in KHT sarcomas.

In summary, delineation of the types of hypoxia in solid tumours is of considerable importance. The presence of acute and/or chronic hypoxia in tumours may have significant impact on the choice of therapies to overcome radiation resistance due to hypoxia. Studies of tumour cell subpopulations may provide valuable information in this area.

These studies were supported by NIH grant CA-36858. The authors wish to thank Dr J. Allalunis-Turner for her discussions and constructive criticisms and B. Granger for the preparation of the manuscript. The excellent technical support of A. Driscoll, S. Morrissey, C. Ostner and K. Wolf is gratefully acknowledged. Dr Steven P. Ellis provided invaluable assistance in the data analysis.

### References

BROWN, J.M. (1979). Evidence for acutely hypoxic cells in mouse tumours and a possible mechanism of reoxygenation. *Br. J. Radiol.*, **52**, 650.

BUSH, R.S., JENKINS, R.D.T., ALLT, W.E.C. & 4 others. (1978). Definitive evidence for hypoxic cells influencing cure in cancer therapy. *Br. J. Cancer*, Suppl. III, **37**, 302.

CHAPLIN, D.J., DURAND, R.E. & OLIVE, P.L. (1985). Cell selection from a murine tumour using the fluorescent probe Hoechst 33342. *Br. J. Cancer*, **51**, 569.

CRISSMAN, A.J. & TOBEY, R.A. (1974). Cell cycle analysis in twenty minutes. *Science*, **184**, 1297.

FRIED, J. & MANDEL, M. (1979). Multi-user system for analysis of data from flow cytometry. *Comput. Prog. Biomed.*, **10**, 218.

GRDINA, D.J., PETERS, L.J., JONES, S. & CHAN, E. (1978). Separation of cells from a murine fibrosarcoma on the basis of size. 1. Relationship between cell size and age as modified by growth *in vivo* or *in vitro*. *J. Natl Cancer Inst.*, **61**, 209.

GRDINA, D.J., SIGDESTAD, C.P. & PETERS, L.J. (1979). Phase specific cytotoxicity *in vivo* of hydroxyurea on murine fibrosarcoma cells synchronized by centrifugal elutriation. *Br. J. Cancer*, **39**, 152.

GUICHARD, M., COURDI, A. & MALAISE, E. (1980). Experimental data on the radiobiology of solid tumours. *J. Europ. Radiother.*, **1**, 171.

HILL, R.P. (1980). An appraisal of *in vivo* assays of excised tumours. *Br. J. Cancer*, **41**, Suppl. IV, 230.

HILL, R.P. & BUSH, R.S. (1977). A new method of determining the fraction of hypoxic cells in a transplantable murine sarcoma. *Radiat. Res.*, **70**, 141.

HILL, R.P., NG, R., WARREN, B.F. & BUSH, R.S. (1979). The effect of intercellular contact on the radiation sensitivity of KHT sarcoma cells. *Radiat. Res.*, **77**, 182.

KENG, P.C., WHEELER, K.T., SIEMANN, D.W. & LORD, E.M. (1981). Direct synchronization of cells from solid tumours by centrifugal elutriation. *Exp. Cell Res.*, **134**, 15.

KENG, P.C., SIEMANN, D.W. & WHEELER, K.T. (1984). Comparison of tumour age response to radiation for cells derived from tissue culture or solid tumours. *Br. J. Cancer*, **50**, 519.

KALLMAN, R.F., SILINI, J. & VAN PUTTEN, L.J. (1967). Factors influencing the quantitation of the *in vivo* survival of cells from solid tumours. *J. Natl Cancer Inst.*, **39**, 539.

MARTIN, W.M.C. & McNALLY, N.J. (1980). The cytotoxic action of adriamycin on tumour cells *in vitro* and *in vivo*. *Br. J. Cancer*, **41**, Suppl. IV, 306.

MEISTRICH, M.L., MEYN, R.E. & BARLOGIE, B. (1977). Synchronization of mouse LP59 cells by centrifugal elutriation separation. *Exp. Cell Res.*, **105**, 169.

OLIVE, P.L., CHAPLIN, D.J. & DURAND, R.E. (1985). Pharmacokinetics, binding and distribution of Hoechst 33342 in spheroids and murine tumours. *Br. J. Cancer*, **52**, 739.

SIEMANN, D.W. (1984). Modification of chemotherapy by nitroimidazoles. *Int. J. Radiat. Oncol. Biol. Phys.*, **10**, 1585.

SIEMANN, D.W. & KENG, P.C. (1984). *In situ* radiation response and oxygen enhancement ratio of KHT sarcoma cells in various phases of the cell cycle. *Br. J. Radiol.*, **57**, 823.

SIEMANN, D.W. & KENG, P.C. (1986). Cell cycle specific toxicity of the Hoechst 33342 stain in untreated or irradiated murine tumor cells. *Cancer Res.*, **46**, 3556.

SIEMANN, D.W. & KOCHANSKI, K. (1981). Combinations of radiation and misonidazole in a murine lung tumor model. *Radiat. Res.*, **86**, 387.

SIEMANN, D.W., LORD, E.M., KENG, P.C. & WHEELER, K.T. (1981). Characterization of cell subpopulations dispersed from solid tumours and separated by centrifugal elutriation. *Br. J. Cancer*, **44**, 100.

SNEDECOR, G.W. & COCHRAN, W.G. (1973). *Statistical methods*. The Iowa State University Press, Ames, Iowa.

STEEL, G.G. (1977). *Growth kinetics of tumours*. Clarendon Press, Oxford.

SUTHERLAND, R.M. & FRANKO, A.J. (1980). On the nature of the radiobiologically hypoxic fraction in tumors. *Int. J. Radiat. Oncol. Biol. Phys.*, **6**, 117.

SUTHERLAND, R.M., EDDY, H.A., BAREHAM, B., REICH, K. & VANANTWERP, D. (1979). Resistance to adriamycin in multicellular spheroids. *Int. J. Radiat. Oncol. Biol. Phys.*, **5**, 1225.

TANNOCK, I. (1978). Cell kinetics and chemotherapy: A critical review. *Cancer Treat. Rep.*, **62**, 1117.

THOMLINSON, R.H. & GRAY, L.H. (1955). The histological structure of some human lung cancers and the possible implications for radiotherapy. *Br. J. Cancer*, **9**, 539.

THOMSON, J.E. & RAUTH, A.M. (1974). An *in vitro* assay to measure the viability of KHT tumour cells not previously exposed to culture conditions. *Radiat. Res.*, **58**, 262.